# Supplementary material for: Impact of Ants on the Order Composition of Canopy Arthropod Communities in Temperate and Tropical Forests
Source: Animals (Basel). 2025 Jun 28;15(13):1914. doi: 10.3390/ani15131914 (PMC12249239; doi:10.3390/ani15131914)
Supplement: Supplementary file 1 [file animals-15-01914-s001.zip › animals-3675379-supplementary.pdf]

Impact of ants on the order composition of canopy arthropod communities in temperate and tropical forests

Andreas Floren [1,2,*] and Tobias Müller [1]

[1]Department of Bioinformatics, Biocentre, University of Würzburg, Am Hubland, D-97074 Würzburg, Germany

[2]Department of Animal Ecology and Tropical Biology, Biocentre, University of Würzburg, Am Hubland, 97074 Würzburg, Germany, E-mail: Telephone: +49 (0)931 31-84376,
Fax: +49 (0)931 31-84352

Supplementary Material

Figure S1 A): To analyze how ants affect arboreal arthropod communities we compared trees dominated by *F. polyctena* ants (Ants) with trees without *F. polyctena* (NoAnts) in temperate forests. The median for trees with "LowAnts" is 16 individuals, the median for "Ants" is 3650 individuals. **B)** The tropical study was conducted in lowland forests on Borneo, Sabah, Malaysia. Since all trees were inhabited by ants, we selected trees with high ant dominance and trees with low ant abundance. The median for the "LowAnts" trees is 366, the median for "Ants" is 3052. Ant abundance was assessed by insecticidal knock down (fogging). The number of trees fogged in both June and August is shown in brackets.

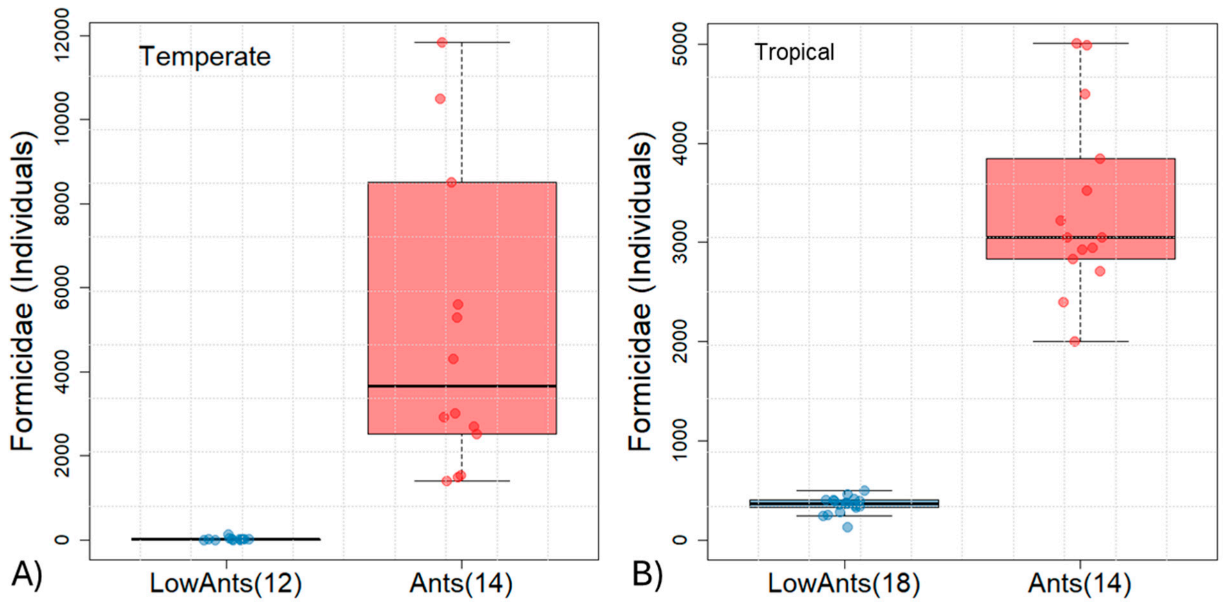

Figure S2: The marginal interaction plots of the logistic regression models show significant higher numbers of arthropods on trees with high ant abundance (Ants) and low ant abundance (NoAnts) across different seasons in temperate forests (above) and tropical forests (below). The interaction term Ants*Season was only significant in the tremperate forests. Significances for the ant impact (A) and the seasonal effect (S) are indicated.

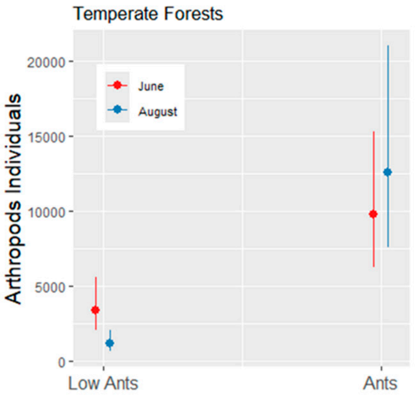

Model:
Total~FacAnts*Season+Sheet, fam=nb()

	Total		
Predictors	*Incidence Rate Ratios*	*CI*	*p*
(Intercept)	2377.57	643.72 – 8781.56	**<0.001**
FacAnts [Ants]	2.88	1.74 – 4.76	**<0.001**
Season [August]	0.35	0.14 – 0.87	**0.023**
Sheet	1.01	0.96 – 1.06	0.658
FacAnts [Ants] × Season [August]	3.65	1.74 – 7.67	**0.001**
Observations	26		
R^2	0.336		

June

	Total		
Predictors	*Incidence Rate Ratios*	*CI*	*p*
(Intercept)	2378.35	884.62 – 6394.27	**<0.001**
FacAnts [Ants]	2.88	1.96 – 4.21	**<0.001**
Sheet	1.01	0.97 – 1.05	0.560
Observations	14		
R^2	0.602		

August

	Total		
Predictors	*Incidence Rate Ratios*	*CI*	*p*
(Intercept)	1.00	1.00 – 1.00	NaN
FacAnts [Ants]	10.49	5.44 – 20.25	**<0.001**
Sheet	1.20	1.18 – 1.21	**<0.001**
Observations	12		
R^2	0.330		

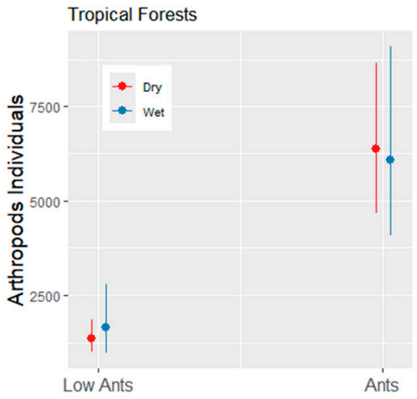

Model:
Total ~ FacAnts*SeasonShort+Sheet+Year,
fam=nb()

	Total		
Predictors	*Incidence Rate Ratios*	*CI*	*p*
(Intercept)	634.94	237.29 – 1698.94	**<0.001**
FacAnts [Ants]	4.68	3.22 – 6.80	**<0.001**
SeasonShort [Wet]	1.21	0.70 – 2.12	0.492
Sheet	1.04	0.99 – 1.08	0.099
Year [1996]	0.94	0.68 – 1.30	0.721
FacAnts [Ants] × SeasonShort [Wet]	0.79	0.36 – 1.74	0.555
Observations	32		
R^2	0.814		

Dry

	Total		
Predictors	*Incidence Rate Ratios*	*CI*	*p*
(Intercept)	235.16	24.32 – 2273.41	**<0.001**
FacAnts [Ants]	4.71	3.19 – 6.95	**<0.001**
Sheet	1.08	0.98 – 1.20	0.122
Year [1996]	0.97	0.66 – 1.43	0.890
Observations	16		
R^2	0.627		

Wet

	Total		
Predictors	*Incidence Rate Ratios*	*CI*	*p*
(Intercept)	951.94	402.20 – 2253.12	**<0.001**
FacAnts [Ants]	3.87	1.56 – 9.60	**0.004**
Sheet	1.03	0.98 – 1.08	0.276
Year [1996]	0.86	0.43 – 1.71	0.662
Observations	16		
R^2	0.925		

Figure S3: A) Modelling Jaccard beta diversity adjusted for spatial autocorrelation by the andonis function. B) Pariwise.adonis was used to compute posthoc-tests.

```
Marginal effects of terms,Number of permutations: 999

adonis2(formula = dmat ~ dist.type + PCoA1 + PCoA2, by = margin")

           Df SumOfSqs      R2      F Pr(>F)
dist.type   6    4.507 0.12510 2.9154  0.001 ***
PCoA1       1    0.659 0.01830 2.5585  0.002 **
PCoA2       1    0.409 0.01135 1.5871  0.017 *
Residual   88   22.672 0.62935
Total      96   36.025 1.00000
```

A)

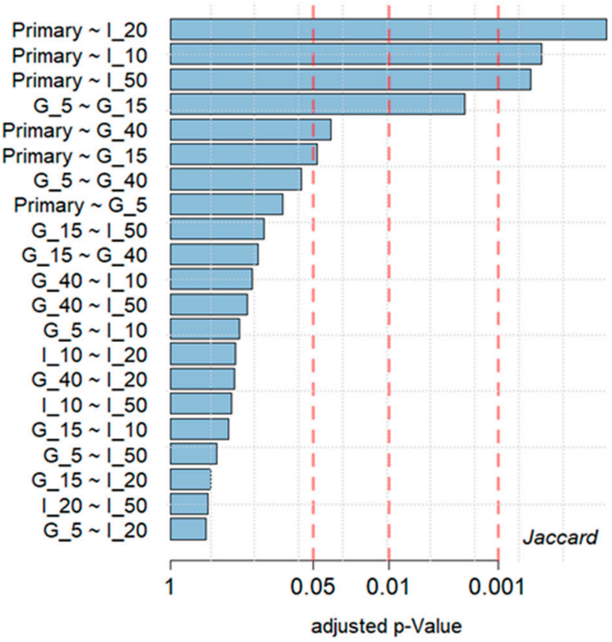

B)
